# Advances in Surgical Management of Malignant Gastric Outlet Obstruction

**DOI:** 10.3390/cancers17152567

**Published:** 2025-08-04

**Authors:** Sang-Ho Jeong, Miyeong Park, Kyung Won Seo, Jae-Seok Min

**Affiliations:** 1 Department of Surgery, Gyeongsang National University Changwon Hospital, Gyeongsang National University College of Medicine, Changwon 51472, Republic of Korea; shjeong@gnu.ac.kr; 2 Department of Anesthesiology, Gyeongsang National University Changwon Hospital, Changwon 51472, Republic of Korea; 3 Department of Surgery, Kosin University Gospel Hospital, Busan 49267, Republic of Korea; 4 Department of Surgery, Korea University College of Medicine, Seoul 02841, Republic of Korea; 5 Division of Foregut Surgery, Korea University Anam Hospital, Seoul 02841, Republic of Korea

**Keywords:** stomach neoplasm, gastric outlet obstruction, endoscopy, surgery, quality of life, palliative management, gastrojejunostomy

## Abstract

Malignant gastric outlet obstruction (MGOO) is a serious complication arising from advanced gastric or pancreatic head cancer, significantly impairing patients’ quality of life by disrupting oral intake and inducing severe gastrointestinal symptoms. With benign causes such as peptic ulcer disease on the decline, malignancies now account for 50–80% of gastric outlet obstruction (GOO) cases globally. This review outlines the pathophysiology, evolving epidemiology, and treatment modalities for MGOO. Therapeutic approaches include conservative management, endoscopic stenting, surgical gastrojejunostomy (GJ), stomach partitioning gastrojejunostomy (SPGJ), and endoscopic ultrasound-guided gastroenterostomy (EUS-GE). While endoscopic stenting offers rapid symptom relief with minimal invasiveness, it has higher rates of re-obstruction. Surgical options like GJ and SPGJ provide more durable palliation, especially for patients with longer expected survival. SPGJ, a modified surgical technique, demonstrates reduced incidence of delayed gastric emptying and may improve postoperative oral intake and survival compared to conventional GJ. EUS-GE represents a promising, minimally invasive alternative that combines surgical durability with endoscopic efficiency, although long-term data remain limited. Treatment selection should consider patient performance status, tumor characteristics, prognosis, and institutional resources. This comprehensive review underscores the need for individualized, multidisciplinary decision-making to optimize symptom relief, nutritional status, and overall outcomes in patients with MGOO.

## Simple Summary

Malignant gastric outlet obstruction (MGOO), mainly caused by advanced gastric or pancreatic cancer, severely impairs quality of life by blocking oral intake and causing gastrointestinal symptoms. With benign causes declining, malignancy now accounts for 50–80% of cases. Treatments include conservative care, endoscopic stenting, surgical gastrojejunostomy (GJ), stomach partitioning GJ (SPGJ), and endoscopic ultrasound-guided gastroenterostomy (EUS-GE). Stenting offers quick relief but carries a high risk of re-obstruction, while GJ and SPGJ provide more durable palliation. SPGJ may improve gastric emptying and survival. EUS-GE is a minimally invasive, promising option, though long-term data are limited. Treatment choice should consider patient condition, tumor status, and institutional capabilities, emphasizing individualized, multidisciplinary care to enhance outcomes.

## 1. Introduction

Malignant gastric outlet obstruction (MGOO) remains a significant clinical challenge that adversely affects patient quality of life, despite advances in gastric cancer treatment and the evolution of various therapeutic strategies [1]. In cases where the cancer is unresectable or recurrent—such as advanced gastric or pancreatic malignancies—effective symptom palliation and improved survival are key treatment goals [2]. MGOO is characterized by mechanical impediment to gastric emptying, typically involving the distal stomach, pylorus, or proximal duodenum. This obstruction leads to symptoms such as nausea, vomiting, early satiety, and abdominal pain, significantly impairing patients’ quality of life [3]. The etiology of gastric outlet obstruction has shifted dramatically in recent decades, with malignant causes now accounting for 50–80% of cases in many regions [1,4].

This review aims to provide a comprehensive synthesis of current surgical and endoscopic options, compare their clinical outcomes, and highlight considerations for selecting optimal intervention strategies in MGOO.

### 1.1. Definition of Gastric Outlet Obstruction (GOO)

Gastric outlet obstruction (GOO) is a clinical syndrome characterized by mechanical blockage at the level of the pylorus or proximal duodenum, leading to symptoms such as postprandial vomiting, epigastric pain, and early satiety. Chronic gastric distension and malnutrition often ensue when normal gastric emptying is impaired [5,6]. Historically, peptic ulcer disease (PUD) was the predominant cause of GOO, but its incidence has significantly declined due to advances in medical therapies, including Helicobacter pylori eradication and widespread use of proton pump inhibitors [4,7]. In recent decades, there has been a significant shift towards malignant etiologies. Common malignant causes include pancreatic cancer (50–60%), gastric cancer (35%), and less frequently, duodenal cancer, cholangiocarcinoma, and metastatic disease [5,8]. This etiological evolution underscores the need for multifaceted treatment strategies that prioritize optimal palliation in advanced disease settings [9].

### 1.2. Epidemiology

The precise global incidence of GOO remains challenging to establish due to heterogeneous study designs and varying definitions of obstruction. The increasing predominance of malignant etiology reflects both the declining prevalence of complicated peptic ulcer disease and the increasing rates of advanced gastrointestinal malignancies. Among malignant etiologies, unresectable gastric cancer contributes to a significant proportion, accounting for up to 35% of GOO cases [5,8]. Pancreatic adenocarcinoma is another major contributor, leading to GOO in approximately 15–25% of patients over the course of their disease. The present shift aligns with broader epidemiological trends in gastrointestinal oncology, where improved early detection methods and advanced oncologic treatments have prolonged patient survival, consequently leading to more cases of locoregionally advanced tumors predisposing to obstruction. A recent study by Govindarajan et al. (2025) reported that GOO is observed in 10–25% of patients with pancreatic and biliary malignancies, highlighting the significant clinical burden of this condition [10]. Furthermore, a national perspective study by Ayesha P Ng et al. (2025) found that among 8186 patients with GOO, 68.4% underwent endoscopic stenting, while 31.6% underwent gastrojejunostomy, indicating the evolving treatment landscape for this condition [11].

### 1.3. Etiology Shift

The transition from benign to malignant etiologies of GOO can be attributed to multiple factors. The effective medical management of peptic ulcer disease, including H. pylori eradication and widespread use of proton pump inhibitors, has significantly decreased the incidence of ulcer-related strictures and complications [4,7]. Concurrently, the global burden of cancer has risen, with gastric and pancreatic cancers remaining among the leading causes of cancer-related mortality. A study to analyze the historical global data by Ju-Li Lin et al. (2024) emphasized that while the overall incidence and mortality of gastric cancer have been decreasing globally, there are still some countries showing an increasing trend, especially among populations younger than 45 years [12]. This evolving pattern underscores the need for tailored surgical and endoscopic interventions for optimal palliation or, in select cases, curative intent. From a therapeutic standpoint, the dominance of malignant GOO necessitates careful patient selection and a multidisciplinary approach. Endoscopic stenting offers rapid symptom relief but may have limited durability. In patients with a longer life expectancy, surgical interventions like gastrojejunostomy or stomach partitioning gastrojejunostomy (SPGJ) may provide a more durable solution while also addressing complications such as delayed gastric emptying [12,13]. The ongoing development of newer techniques, such as endoscopic ultrasound-guided gastroenterostomy (EUS-GE), further exemplifies how innovations are reshaping the treatment landscape for malignant GOO. A recent study by Khoi Van Tran et al. (2024) compared EUS-GE with duodenal stenting for malignant GOO, demonstrating promising outcomes for this novel approach [14]. These advancements highlight the importance of continued research and innovation in addressing the changing epidemiology and etiology of GOO.

### 1.4. Evidence Grading

To assess the quality of evidence supporting clinical recommendations within this study, we employed an evidence grading approach based on the GRADE (Grading of Recommendations Assessment, Development, and Evaluation) framework [15]. This method systematically evaluates the certainty of evidence by considering study design, risk of bias, consistency and precision of results, directness of evidence, and potential publication bias. Observational studies, case series, and expert opinions were generally graded as low to moderate quality, while randomized controlled trials (RCTs), when available, were rated as high quality. Given the predominance of observational data in this field, grading was undertaken with caution, and the strength of recommendations was aligned accordingly. The grading process was conducted independently by two reviewers, with discrepancies resolved through discussion to ensure reliability and transparency.

## 2. Etiology of Gastric Outlet Obstruction

Gastric outlet obstruction (GOO) encompasses a broad spectrum of both malignant and benign conditions. While improvements in medical management have reduced certain benign causes over time, the global prevalence of malignancies has led to a rise in malignant GOO. Accurate identification of the specific etiology is crucial in determining the most appropriate intervention—ranging from endoscopic stenting to surgical bypass.

### 2.1. Malignant Causes

Malignant causes now represent a growing proportion of GOO cases, reflecting both a decline in complications from peptic ulcer disease and a rising incidence of advanced gastrointestinal malignancies. Among these, gastric cancer accounts for up to 35% of malignant GOO cases, most often presenting at a locally advanced or metastatic stage [6]. Pancreatic adenocarcinoma contributes to approximately 15–25% of cases, typically through direct invasion or external compression of the gastric outlet [3]. Other neoplasms, such as gastric lymphoma, duodenal or ampullary tumors, and malignancies of the gallbladder or bile ducts, may also lead to obstruction by infiltrating or compressing adjacent gastrointestinal structures.

Managing malignant GOO poses distinct clinical challenges. Endoscopic stenting offers prompt symptom relief but is frequently associated with a higher need for reintervention. In contrast, surgical options—such as conventional gastrojejunostomy (GJ) or stomach partitioning GJ—tend to provide more durable palliation, albeit with increased procedural risk [14].

### 2.2. Benign Causes

Although MGOO is increasingly common, several benign conditions remain important causes. Peptic ulcer disease (PUD), once a leading etiology, can still result in obstruction due to chronic inflammation and fibrosis [3]. Chronic pancreatitis may cause external compression of the gastric outlet via fibrosis or pseudocysts, while Crohn’s disease can lead to duodenal strictures. Corrosive injuries from caustic ingestion may result in long-term scarring and luminal narrowing [16]. Less frequent causes include gastrointestinal tuberculosis, percutaneous endoscopic gastrostomy tube complications, congenital anomalies such as annular pancreas, and intramural hematomas. Gastric bezoars and volvulus may also lead to mechanical obstruction [17].

The incidence of PUD-related GOO has declined with proton pump inhibitor use and Helicobacter pylori eradication [6]. However, distinguishing benign from malignant causes remains critical, as treatment strategies differ and may involve endoscopic or surgical approaches depending on the etiology.

## 3. Clinical Manifestations

Gastric outlet obstruction (GOO) typically presents with a characteristic constellation of upper gastrointestinal symptoms stemming from impaired gastric emptying. Early recognition of these symptoms is crucial for prompt diagnosis and treatment, as prolonged obstruction can lead to significant nutritional deficits and electrolyte imbalances [18,19].

• Epigastric Pain
  Epigastric pain is indeed a common initial complaint in patients with GOO, and its pathophysiology is closely linked to gastric distension and irritation [7,20]. The severity of pain may fluctuate depending on the degree of obstruction and the underlying cause—malignant or benign.
• Nausea and Vomiting
  Nausea and vomiting, particularly postprandial vomiting, are hallmark features of GOO [6,20]. Accumulated gastric contents cannot pass through the obstructed pylorus or duodenum, leading to retching and eventual vomiting of undigested food [7]. These symptoms can be especially distressing and may progressively worse if the obstruction persists [7,20].
• Early Satiety
  Patients frequently report a sensation of fullness or early satiety after minimal food intake. This occurs because even a small volume of food can exacerbate gastric distension behind the obstruction, preventing normal accommodation and peristalsis [21,22].
• Abdominal Distension
  Chronic accumulation of gastric contents and gas contributes to visible abdominal distension or bloating. Physical examination may reveal a percussion splash, reflecting significant fluid retention within the stomach. Such findings underscore the mechanical nature of GOO and help differentiate it from functional dyspepsia [22,23,24].
• Weight Loss
  Inadequate oral intake, combined with persistent vomiting, often leads to weight loss and compromised nutritional status. This issue is especially pronounced in malignant GOO, where advanced tumor burden may further diminish appetite. Early nutritional interventions—such as parenteral or enteral feeding—can be critical to maintaining the patient’s overall condition [25].

## 4. Diagnosis

Accurate diagnosis of gastric outlet obstruction (GOO) relies on a combination of clinical evaluation, imaging modalities, and endoscopic assessment. This multi-pronged approach is crucial for determining the underlying etiology—whether malignant or benign—and guiding appropriate therapeutic strategies [4].

### 4.1. History & Physical Examination

(1) Symptom Assessment
  A thorough patient history is essential to characterize the nature and onset of symptoms, such as postprandial vomiting, weight loss, and the presence or absence of epigastric pain [7,20]. Specific inquiry into the volume, timing, and content of vomitus can help localize the level of obstruction.
(2) Nutritional Status and Hydration
  Chronic vomiting and inadequate oral intake often result in electrolyte imbalances and malnutrition. Clinicians should evaluate weight changes, muscle wasting, and signs of dehydration [26].
(3) Physical Examination
  On physical exam, a “succussion splash,” a sloshing sound heard during abdominal movement, may be detected in the epigastrium, suggesting fluid retention within a distended stomach [7,20]. Abdominal distension or tenderness could further point to possible tumor masses or secondary complications like peritonitis [27].

### 4.2. Endoscopic Evaluation

**Figure 1:**
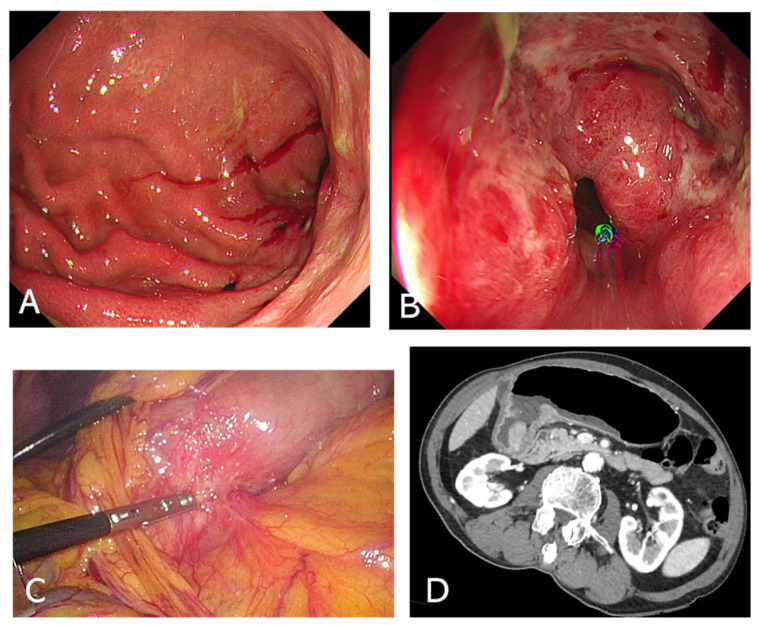
Endoscopic (**A**,**B**), surgical (**C**) findings, and computed tomography image (**D**) of malignant gastric outlet obstruction.

- Upper Endoscopy (EGD)
  Endoscopic examination is vital for direct visualization of the gastric outlet and duodenum (Figure 1A,B). It confirms the presence of an obstructing lesion and enables biopsy for histopathological analysis, distinguishing between benign and malignant etiologies. In many cases, EGD also offers therapeutic potential, such as stent placement [28].
- Differential Diagnosis
  Endoscopic findings help rule out peptic ulcer–related strictures, malignancies like gastric cancer or lymphoma, or more unusual causes such as impacted bezoars. If malignancy is suspected, obtaining multiple biopsies from the lesion edge and surrounding mucosa can improve diagnostic accuracy [29].

### 4.3. Imaging Studies

- CT Scan
  Contrast-enhanced computed tomography (CT) of the abdomen is a key diagnostic tool, allowing for visualization of gastric distension, fluid levels, and the precise location of the obstruction (Figure 1D). It also helps identify extrinsic masses or lymph node involvement in malignant cases [30].
- Additional Imaging Considerations
  ➀ Plain Abdominal X-ray: May show an enlarged, fluid-filled stomach with air-fluid levels [31].
  ➁ MRI: Occasionally used in complex cases requiring detailed soft-tissue evaluation or in patients with contraindications to CT contrast media [32].

## 5. Treatment Strategies for Malignant GOO (Figure 2)

Malignant Gastric Outlet Obstruction (MGOO) is a clinical condition where the pylorus or duodenum is mechanically obstructed due to advanced gastric or pancreatic cancer, making adequate oral intake difficult. This significantly impacts patient survival and quality of life. The main treatment strategies include:

**Figure 2:**
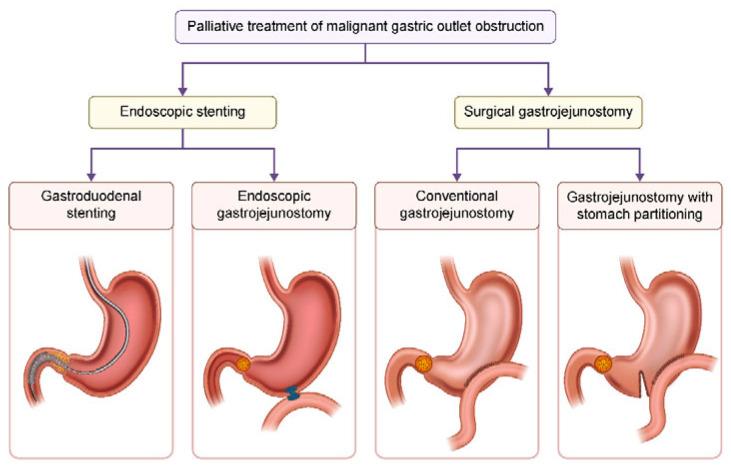
Treatment modalities for palliation of malignant gastric outlet obstruction (MGOO, Malignant Gastric Outlet Obstruction).

### 5.1. Conservative Management [5]

Conservative management for patients with malignant gastric outlet obstruction includes the following elements:

• Intravenous fluid administration and correction of electrolyte imbalances
• Maintaining nil per os (NPO) status
• Administration of high-dose proton pump inhibitors to reduce gastric secretions
• Nasogastric tube insertion when necessary

These conservative approaches are crucial for stabilizing the patient’s overall condition and serve as a preparatory stage for potential interventional treatments.

• Evidence Quality: Low to moderate—these recommendations are largely supported by clinical consensus and observational studies rather than randomized controlled trials (RCTs). They represent standard supportive care derived from physiological rationale and clinical experience.
• Grading: Grade C (based on observational data and expert opinion). Essential but not directly evidence-proven interventions.

### 5.2. Endoscopic Stenting [33]

• Provides rapid symptom relief and shorter hospital stays [34,35].
• Higher risk of re-obstruction due to tumor ingrowth, potentially requiring repeat procedures.
• Suitable for patients with shorter life expectancy.
• Evidence Quality: Moderate—based mostly on observational cohort studies and some comparative analyses; a few prospective studies but limited RCT evidence. Benefits in symptom palliation are consistently reported though long-term patency and reintervention rates vary.
• Grading: Grade B (moderate quality observational evidence). Well-supported for palliation but with known limitations.
• Patient Selection Criteria:
• Inclusion:
  - Patients with unresectable malignant gastric outlet obstruction requiring palliation.
  - Poor surgical candidates due to comorbidities or limited life expectancy (generally <3–6 months).
  - Patients needing rapid symptom control with minimal invasiveness.
• Exclusion:
  - Patients with extensive tumor infiltration precluding safe stent deployment.
  - Those with high risk of stent-related complications or expected survival longer than 6 months where durable surgical options may be preferable.
• Long-term Outcomes
  - Technical and clinical success rates are high, but stent patency is typically limited; re-obstruction and need for reintervention are common within months [10,36].
  - Quality of life improves immediately after stent placement, but benefits are often transient as restenosis or obstruction may recur within 6 months [10,36].
• Survival
  - Median survival after stenting is generally about 2 months in advanced cases, though selected patients (such as those with unresectable pyloric adenocarcinoma) can have survival exceeding 1 year [10,36,37].
• Quality of Life
  - Symptom palliation is rapid and marked by improved ability to tolerate oral intake. However, repeated interventions due to obstruction limits sustained gains in quality of life.

### 5.3. Conventional Surgical Gastrojejunostomy [38]

• Traditional standard treatment offering long-term symptom relief.
• Higher risk of postoperative complications and longer hospital stays.
• Recommended for operable patients with a life expectancy of 3–6 months or more.
• Evidence Quality: Moderate—supported by retrospective case series and some prospective observational studies comparing outcomes with stenting. RCTs are limited due to ethical/practical concerns.
• Grading: Grade B (moderate observational evidence). Long-term outcome benefit acknowledged; perioperative risks well characterized.
• Patient Selection Criteria:
• Inclusion:
  - Patients who are operable with acceptable performance status (e.g., ECOG 0–2).
  - Life expectancy ≥3–6 months where longer-lasting symptom relief justifies surgery risk.
• Exclusion:
  - Patients with prohibitive surgical risk due to comorbidities or poor performance status.
  - Very limited life expectancy (<3 months) where surgical morbidity outweighs benefit.
• Long-term Outcomes
  - Offers more durable symptom relief than stenting, with lower rates of reintervention and delayed gastric emptying in the long term [1,39,40].
• Survival
  - The median survival is longer than with stenting, typically reported at 2.6–2.7 months for stenting versus about 3.5–4 months for GJ, though some meta-analyses and studies report longer survival (up to 7–8 months) for surgical candidates [38,39].
• Quality of Life
  - Provides sustained improvement in oral intake and nutritional status [40]. There is no significant quality-of-life difference compared to endoscopic interventions, but surgical GJ may reduce the need for repeated procedures [38].

### 5.4. Stomach Partitioning Gastrojejunostomy (SPGJ) [41]

• SPGJ is a surgical technique developed to address the limitations of conventional gastrojejunostomy.
• It partially divides the stomach, allowing food to be directly evacuated into the jejunum.
• It reduces the incidence of delayed gastric emptying (DGE).
• It decreases the risk of tumor-related bleeding.
• Technical detail
  - The SPGJ technique involves partial division of the stomach at the junction of the gastric body and antrum, or approximately 5 cm proximal to the upper margin of the tumor, using a linear stapler. This maneuver preserves a narrow 2–3 cm strip of gastric corpus adjacent to the lesser curvature, ensuring vascular continuity and physiologic drainage of gastric secretions. An enterotomy is then made at the greater curvature of the proximal stomach, and a corresponding opening is created on the mesenteric border of the jejunum, situated 5–10 cm distal to the ligament of Treitz. The jejunal limb is subsequently brought to the gastric anastomotic site posterior to the transverse colon, and a side-to-side gastrojejunostomy is fashioned using a linear stapler. The common enterotomy is closed by continuous, barbed suture, completing the anastomosis [34].
• Advantages of SPGJ:
  ➀ Faster recovery of oral intake compared to conventional gastrojejunostomy
  ➁ More complete diet possible after 15 days
  ➂ Reduced incidence of DGE
  ➃ Decreased need for reoperation
  ➄ Potential for improved overall survival rates

Recent studies have shown that SPGJ demonstrates similar surgical outcomes to endoscopic stenting while offering a higher likelihood of solid food intake [42].

In conclusion, SPGJ represents a safe and effective treatment option for patients with malignant gastric outlet obstruction. It is particularly useful as an alternative when endoscopic ultrasound-guided gastroenterostomy (EUS-GE) is not feasible or in resource-limited healthcare settings [41].

• Evidence Quality: Low to moderate—mostly from small cohort studies, retrospective analyses, and single-center experiences; comparative data limited and lacking large RCTs.
• Grading: Grade C to B (primarily observational studies). Promising but requires further well-designed studies to confirm benefits and generalizability.
• Patient Selection Criteria:
• Inclusion:
  - Patients with good functional status (e.g., ECOG 0–1) and operable tumor burden.
  - Expected survival longer than 3–6 months to benefit from improved nutritional recovery.
  - Cases where EUS-GE is not available or contraindicated.
• Exclusion:
  - Patients with poor performance status or significant comorbidities precluding surgery.
  - Advanced tumor invasion limiting feasibility of stomach partitioning.
• Long-term Outcomes
  - Associated with lower rates of delayed gastric emptying, vomiting, and prokinetics requirement than conventional GJ [34,35].
  - Patients tolerate solid diet earlier and have shorter postoperative hospital stay [34,35,43].
• Survival
  - No significant difference in survival compared to conventional GJ; the ability to eat a full diet and receive subsequent chemotherapy are more strongly associated with survival than the surgical technique itself [35,43].
• Quality of Life
  - SPGJ provides a better and faster return to normal diet, which may translate to improved quality of life in the early and medium term [43].

### 5.5. EUS-Guided Gastroenterostomy [44]

• A novel technique combining the advantages of surgery and endoscopy.
• Minimally invasive with potential for long-term efficacy.
• Not yet standardized and requires further research.
• Evidence Quality: Low—mainly early-phase studies, pilot cohorts, and case series. Lack of standardization and robust comparative data.
• Grading: Grade C (low-quality evidence). Promising but investigational; further rigorous trials needed.
• Patient Selection Criteria:
• Inclusion:
  - Patients deemed high-risk for conventional surgery but with longer expected survival than palliation candidates.
  - Those in centers with experienced endoscopists and availability of specialized equipment.
  - Patients unsuitable for stenting due to anatomical factors or prior stent failure.
• Exclusion:
  - Patients in resource-limited settings without access to advanced endoscopic modalities.
  - Unfit patients who cannot tolerate longer procedural times or potential complications.
  - Lack of institutional expertise.
• Long-term Outcomes
  - High technical and clinical success rates (often >90%) [45,46,47].
  - EUS-GE demonstrates lower recurrence and reintervention rates compared to duodenal stenting, and comparable long-term patency and adverse event rates to surgery [46].
• Survival
  - Overall survival is similar to surgical and stent-based approaches; however, fewer reinterventions and improved tolerance of oral intake are strengths of EUS-GE [46,48].
• Quality of Life
  - Better patient-reported eating habits and sustained oral intake improvement compared to duodenal stenting [46].
  - Quality of life scores at 1 month are similar to surgery but EUS-GE can reduce hospital stay and frequency of intervention [48].

### 5.6. Comparative Features of GJ, SPGJ, and EUS-GE [48,49,50,51,52,53]

Table 1 summarizes the comparative clinical and procedural characteristics of gastrojejunostomy (GJ), stomach partitioning gastrojejunostomy (SPGJ), and endoscopic ultrasound-guided gastroenterostomy (EUS-GE). The incidence of delayed gastric emptying (DGE) was notably higher in patients who underwent conventional GJ, with rates reported between 26% and 43.6%, whereas both SPGJ and EUS-GE demonstrated significantly lower DGE rates (2.1–6.7% and similarly low, respectively). Recovery of oral intake was relatively faster in patients receiving SPGJ or EUS-GE compared to those undergoing GJ. From a technical perspective, SPGJ and EUS-GE were associated with higher procedural complexity than GJ. In terms of long-term patency, while GJ showed good outcomes, SPGJ exhibited superior results, and EUS-GE has shown promising long-term patency in recent studies. Regarding patient selection, GJ was generally suitable for a wide range of patients, SPGJ was more appropriate for those with a good performance status, and EUS-GE was typically performed in specialized centers with the requisite expertise and equipment.

• Evidence Quality: Low to moderate—based on pooled observational data, retrospective comparisons, and limited prospective cohorts. Direct head-to-head RCTs rare or absent.
• Grading: Grade C to B. The comparative advantages described should be interpreted cautiously in light of study heterogeneity and potential biases.

## 6. Considerations for Treatment

### 6.1. Laparoscopic vs. Open Surgery

Laparoscopic gastrojejunostomy (LGJ) and open gastrojejunostomy (OGJ) are two distinct surgical approaches employed in the management of malignant gastric outlet obstruction. These techniques differ significantly in terms of surgical method, outcomes, and clinical applicability. From a technical standpoint, LGJ is performed using a minimally invasive approach involving several small abdominal incisions, through which a laparoscope and specialized instruments are inserted. In contrast, OGJ requires a single large incision in the abdominal wall, providing direct access to the operative field.

In terms of surgical outcomes, LGJ offers several advantages over OGJ. Patients undergoing LGJ generally experience less postoperative pain, improved cosmetic outcomes due to smaller incisions, and a reduced risk of developing incisional hernias. Furthermore, LGJ is associated with a shorter hospital stay. However, these benefits come at the cost of a longer operative time (average 76.8 min for LGJ vs. 55.8 min for OGJ) and increased technical difficulty, which may limit its widespread adoption in some clinical settings [54].

Regarding complications, both LGJ and OGJ demonstrate comparable overall complication rates. Nonetheless, LGJ has been associated with a significantly lower incidence of delayed gastric emptying, reported as 0% compared to 26.1% in patients undergoing OGJ [55,56]. In terms of postoperative recovery, patients receiving LGJ typically resume oral intake earlier (median of 2 days versus 4 days in OGJ) and generally exhibit a smoother recovery trajectory [55,57].

With respect to clinical applicability, LGJ can be safely performed even in patients with a history of upper abdominal surgery, expanding its utility in selected cases [4,56,58]. Despite these advantages, OGJ remains the more commonly utilized procedure, likely due to its relative simplicity and broader surgeon familiarity.

In conclusion, while LGJ is a less invasive alternative to OGJ that offers enhanced recovery and superior cosmetic results, it is associated with longer operative times and greater technical demands. Both approaches yield similar overall complication profiles. Therefore, the choice between LGJ and OGJ should be individualized based on patient-specific factors and the operating surgeon’s experience [59].

### 6.2. Stomach Partitioning Gastrojejunostomy (SPGJ) vs. Conventional GJ

Stomach partitioning gastrojejunostomy (SPGJ) and conventional gastrojejunostomy (CGJ) differ significantly in several aspects.

First, in terms of surgical technique, SPGJ involves partial division of the stomach, allowing food to be directly evacuated into the jejunum, whereas CGJ simply connects the stomach to the jejunum [42].

The incidence of delayed gastric emptying (DGE) is significantly lower in SPGJ, ranging from 2.1% to 6.7%, compared to the relatively higher rate of 26% to 43.6% in CGJ [13].

Oral intake recovery is also faster in SPGJ, with patients being able to resume a more complete diet within 15 days postoperatively, whereas CGJ is associated with a relatively slower recovery [34,60].

Regarding the flow of gastric contents, approximately 95% of the gastric contents are evacuated through the anastomosis in SPGJ. In contrast, gastric contents in CGJ tend to flow toward the pylorus, which may contribute to delayed emptying [34].

Hospital stay is shorter in SPGJ, with an average of 7 days, compared to 9 days in CGJ [34,60].

In terms of survival outcomes, SPGJ is associated with a tendency toward longer overall survival, with an average duration of 189.2 days, while CGJ shows a relatively shorter survival period, averaging 115.2 days [61].

### 6.3. Stomach Partitioning: Pros & Cons

The partition of stomach procedures, such as stomach partitioning gastrojejunostomy (SPGJ), presents a number of advantages as well as limitations when compared to conventional gastrojejunostomy (CGJ).

Among the primary advantages, SPGJ is associated with a significantly lower incidence of delayed gastric emptying (DGE), with reported rates ranging from 2.1% to 6.7%, in contrast to the 26% to 43.6% observed in CGJ [13,43].

In terms of postoperative nutritional recovery, patients undergoing SPGJ are more likely to resume a normal diet, with 96% achieving this milestone compared to 72% in the CGJ group [43]. Moreover, a more complete diet is typically achievable within 15 days following surgery in SPGJ patients [62].

Gastric emptying function also appears to be enhanced with SPGJ. Numerical simulations have demonstrated increased fluid flow throughout most regions of the stomach, which facilitates more efficient gastric emptying [34]. Additionally, some clinical studies suggest a trend toward improved overall survival in patients treated with SPGJ, with reported survival durations averaging 189.2 days, compared to 115.2 days for CGJ [60]. Another potential benefit of SPGJ is the reduction in tumor bleeding, achieved by minimizing direct contact between food and the tumor [61].

Despite the notable advantages of SPGJ over CGJ in treating gastric outlet obstruction, SPGJ also has several limitations [13,34,63]. Technically, it is a more complex procedure than CGJ and generally requires a higher level of surgical expertise. The increased complexity also contributes to a longer operation time due to the additional procedural steps involved. Although the rate is not significantly elevated, the complexity of the reconstruction in SPGJ may lead to an increased risk of anastomotic complications.

Furthermore, as a relatively newer technique, SPGJ lacks extensive long-term outcome data, and potential late complications are not as well characterized as those associated with CGJ. Lastly, SPGJ may not be suitable for all patients, particularly those with severely impaired performance status or a very limited life expectancy, for whom the invasiveness of the procedure may outweigh its potential benefits.

## 7. Factors to Consider in Treatment Selection

When determining the most appropriate treatment for malignant gastric outlet obstruction, a comprehensive evaluation of multiple factors is essential (Figure 2).

**Figure 3:**
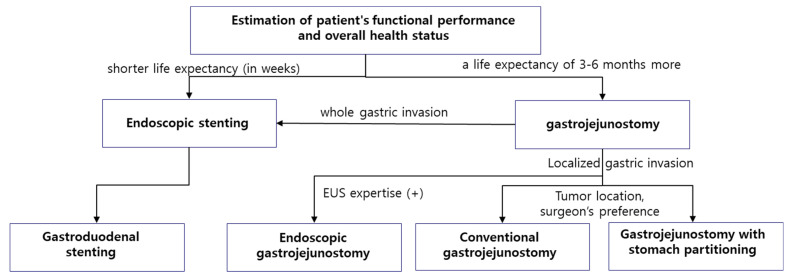
Determining the appropriate treatment for patients with gastric outlet obstruction.

Foremost among these is the patient’s functional performance and overall health status. A patient’s physical condition and ability to tolerate invasive procedures are critical in guiding treatment choices. The stage of the cancer and overall prognosis must also be considered, as patients with a longer life expectancy—typically greater than two to three months—are more likely to benefit from surgical interventions [64] (Figure 3).

Tumor characteristics, including its location, size, and resectability, play an important role in determining whether an endoscopic or surgical approach is more suitable. Nutritional status is another key consideration, as malnutrition can impair a patient’s ability to endure and recover from treatment [4].

The presence of additional complications, such as biliary obstruction, may necessitate modifications to the treatment strategy [65]. Furthermore, the availability of expertise and institutional resources—for example, trained personnel and equipment for advanced procedures such as endoscopic ultrasound-guided gastroenterostomy—can influence what options are feasible [10].

Nutritional status is another critical factor, particularly because patients with malignant GOO frequently present with severe malnutrition due to reduced oral intake and tumor-related catabolism. Nutritional assessment and intervention should be initiated at admission, ideally under the guidance of a clinical nutritionist. Evidence suggests that proper nutritional support not only improves general health but also reduces the risk of anastomotic dehiscence and postoperative complications, even in palliative settings [66]. Nutritional care should be an integral part of the multidisciplinary treatment planning.

Another consideration is the management of biliary obstruction, which frequently coexists in patients with advanced periampullary, pancreatic, or biliary malignancies. Although internal biliary stenting may be attempted, particularly via endoscopic retrograde cholangiopancreatography (ERCP), this approach is often unfeasible due to tumor infiltration or altered anatomy. In such cases, percutaneous external biliary drainage remains the most reliable method for relieving cholestasis and improving liver function prior to surgical or palliative interventions. Timely recognition and management of biliary obstruction are essential for optimizing outcomes and facilitating subsequent treatments, including chemotherapy and surgery [67,68,69,70].

Cost-effectiveness is one of the very important factors in procedure selection. Some studies indicate that EUS-GE tends to be the most cost-effective approach due to its significantly lower total and procedural costs compared with surgical methods, attributed to shorter hospital stays, minimally invasive technique, and fewer needs for reintervention [71,72]. GJ, while incurring higher initial and overall costs because of longer operative time and hospital admission, is justified in patients with longer expected survival as it provides sustained symptom relief; its cost-effectiveness improves in this patient subset despite greater upfront expense. SPGJ, although dedicated cost-effectiveness analyses are sparse, demonstrates hospital and procedural costs comparable to conventional GJ, while offering the clinical benefit of earlier oral intake recovery without added expenses, suggesting no economic disadvantage over GJ. Therefore, EUS-GE is generally the most economically favorable option, especially in centers with expertise, while GJ is most cost-effective for those with prolonged survival, and SPGJ offers early postoperative advantages with similar resource utilization to GJ.

Patient preferences and quality of life considerations should not be overlooked; aligning treatment with the patient’s values and desired outcomes is paramount [10]. Additionally, the chosen intervention should, when possible, preserve the ability to administer future palliative chemotherapy if clinically indicated.

Though not a primary determinant, the cost-effectiveness and resource utilization of different treatment modalities may be considered, especially in settings with constrained healthcare resources [65]. Lastly, each treatment option must be assessed for its associated risks, including the likelihood of complications and the potential need for reintervention.

## 8. Conclusions

The management of malignant gastric outlet obstruction requires an individualized, multidisciplinary approach. Endoscopic stenting remains ideal for patients with poor performance status and limited survival. Surgical CGJ and SPGJ offer superior long-term outcomes and are better suited for patients with longer prognoses compared with endoscopic stenting. EUS-GE presents a promising alternative, pending further validation. Continued innovation and evidence generation will be essential to refine therapeutic algorithms and improve patient-centered outcomes.

## Figures and Tables

**Table 1:** Comparative Features of GJ, SPGJ, and EUS-GE.

Feature	GJ	SPGJ	EUS-GE
DGE Rate	High (26–43.6%)	Low (2.1–6.7%)	Low to intermediate (needs more data)
Oral Intake Recovery	Moderate	Faster	Moderate to fast
Technical Complexity	Moderate	Higher than GJ	High (requires EUS expertise)
Long-Term Patency	Good	Better	Promising (needs more data)
Suitability	General	Good performance status	Specialized centers (requires EUS setup)
Procedure time (minutes)	90–170	90–150	35–96
Hospital stay (days)	9–10	7–9	2–7

GJ, gastrojejunostomy; SPGJ, stomach partitioning gastrojejunostomy; EUS-GE, endoscopic ultrasound guided gastroenterostomy; DGE, delayed gastric emptying.
